# Determinants Influencing the Adoption of Internet Health Care Technology Among Chinese Health Care Professionals: Extension of the Value-Based Adoption Model With Burnout Theory

**DOI:** 10.2196/37671

**Published:** 2023-03-10

**Authors:** Dongsheng Bian, Yuyin Xiao, Keyu Song, Minye Dong, Li Li, Ross Millar, Chenshu Shi, Guohong Li

**Affiliations:** 1 School of Public Health Shanghai Jiao Tong University School of Medicine Shanghai China; 2 Department of Clinical Nutrition Ruijin Hospital Shanghai Jiao Tong University School of Medicine Shanghai China; 3 China Hospital Development Institute Shanghai Jiao Tong University Shanghai China; 4 Health Services Management Centre University of Birmingham Birmingham United Kingdom; 5 Center for Health Technology Assessment China Hospital Development Institute Shanghai Jiao Tong University Shanghai China

**Keywords:** internet health care technology, value-based adoption model, employee burnout, China, health care, health care workers, internet, technology, cross-sectional study, burnout, internet hospitals

## Abstract

**Background:**

The global COVID-19 pandemic has been widely regarded as a catalyst for adopting internet health care technology (IHT) in China. IHT consists of new health care technologies that are shaping health services and medical consultations. Health care professionals play a substantial role in the adoption of any IHT, but the consequences of doing so can often be challenging, particularly when employee burnout is prevalent. Few studies have explored whether employee burnout influences the adoption intention of IHT in health care professionals.

**Objective:**

This study aims to explain the determinants influencing the adoption of IHT from the perspective of health care professionals. To do so, the study extends the value-based adoption model (VAM) with consideration for employee burnout as a determining factor.

**Methods:**

A cross-sectional web-based survey using a sample of 12,031 health care professionals selected through multistage cluster sampling from 3 provinces in mainland China was conducted. The hypotheses of our research model were developed based on the VAM and employee burnout theory. Structural equation modeling was then used to test the research hypotheses.

**Results:**

The results indicate that perceived usefulness, perceived enjoyment, and perceived complexity positively correlate with perceived value (β=.131, P=.01; β=.638, P<.001; β=.198, P<.001, respectively). Perceived value had a positive direct effect on adoption intention (β=.725, P<.001), perceived risk negatively correlated with perceived value (β=−.083, P<.001), and perceived value negatively correlated with employee burnout (β=−.308, P<.001). In addition, employee burnout was negatively related to adoption intention (β=−.170, P<.001) and mediated the relationship between perceived value and adoption intention (β=.052, P<.001).

**Conclusions:**

Perceived value, perceived enjoyment, and employee burnout were the most important determinants of IHT adoption intention by health care professionals. In addition, while employee burnout was negatively related to adoption intention, perceived value inhibited employee burnout. Therefore, this study finds that it is necessary to develop strategies to improve the perceived value and reduce employee burnout, which will benefit the promotion of the adoption intention of IHT in health care professionals. This study supports the use of the VAM and employee burnout in explaining health care professionals’ adoption intention regarding IHT.

## Introduction

During the past several years, the Chinese government has been committed to promoting the use of the internet to provide health care services. Internet health care has experienced several major development phases with the help of the “internet plus” national policy in 2015 [1]. The COVID-19 pandemic brought in a second phase by rapidly catapulting telemedicine into the forefront of health care delivery [2]. These measures have been promoted in optimizing the allocation of medical resources, alleviating the high cost of medical treatment, and promoting the independence and well-being of society. Internet hospitals are a new health care service facilitated by technology. Unlike traditional health services, such hospitals rely on the development of emerging technologies, including cloud computing, big data, and artificial intelligence [3]. These hospitals provide web-based diagnosis and treatment, web-based drug purchase, disease management, and other health care services. A raft of research has been undertaken regarding such developments, with patients’ attitudes and perceptions of internet health care well documented [4-6]. However, as providers of medical services, health care professionals’ understanding and attitudes toward internet technology have been underexplored.

Internet health care technology (IHT) is defined as “a form of new healthcare technologies in terms of professional health services and medical consultations” [7,8]. It represents “noncontact medicine” where instead of meeting in person, health care professionals and patients can exchange information on the internet through text, pictures, voice, and other means. IHT has developed rapidly in recent years with multiple modes of applications, such as telemedicine, teleconsultation, telerehabilitation, internet health care, digital health care, eHealth, and Internet of Medical Things [9-13]. The benefits of IHT include the promotion of resource optimization, the improvement of medical hardware and software, the humanization of medical service models, the proximity of medical costs, and the improvement of the efficiency and accuracy of doctors’ diagnoses. Besides, the IHT represents services provided by health care professionals in general, including doctors, nurses, pharmacists, and others [14].

A growing number of Chinese health care professionals are providing internet health care services via internet hospitals; among them, the majority are full-time employees of Chinese public hospitals [15]. It has been documented how the constant switch between online and offline channels has led to problems with increased workload [16]. For health care professionals, IHT requires them to learn and update new and additional technologies and practices. In addition, employee burnout (BUR) is a prevalent issue, with patients of burned-out health care professionals experiencing more errors and having lower satisfaction with their care [17,18]. Attention to such an area has great importance particularly as physician burnout, characterized by emotional exhaustion or depersonalization, is such a widespread issue [19]. Wen et al [20] reported that 76.9% of Chinese physicians reported burnout symptoms. The COVID-19 pandemic has added new social and job-related factors that increase the risk of burnout in health care professionals worldwide [21-24]. Otherwise, Shah et al [25] found that although teledermatology has become more prevalent as a result of the pandemic, 37% of physicians reported teledermatology as a contributor to their professional burnout. Gardner et al [26] reported that health information technology–related stress in physicians is common and associated with burnout symptoms. Bambe [27] found that increased use of technology could also lead to BUR. Shanafelt et al [28] reported that physicians who used electronic health records or computerized physician entry had higher rates of burnout. Kroth et al [29] showed that data entry requirements, inefficiently designed user interfaces, and information overload brought on by technology were associated with physician stress.

A variety of factors are associated with the adoption of new technology (see Multimedia Appendix 1 [7,8,14,30-78] for more details about literature review and hypotheses development). Further research is needed to determine if the development of IHT can improve health care services or if it will simply increase workloads and even aggravate burnout. Few studies have explored factors affecting the willingness to use IHT from the perspective of health care professionals, considering the impact of BUR. To achieve this goal, a measurement instrument based on the unified theory of the value-based adoption model (VAM) and BUR was used. The interrelationships between the BUR and adoption intention (AI) are investigated with the view to providing an enhanced understanding of factors that may influence the willingness to accept IHT from the perspective of health care professionals.

## Methods

### Development of the Study Questionnaire

A survey questionnaire was developed for this study. The questionnaires contained a sociodemographic section (age, sex, education background, and professional title), BUR, and the VAM model (see Multimedia Appendix 1 for more details about literature review and hypotheses development).

#### VAM Model

A survey questionnaire informed by the VAM was developed. This model seeks to measure the perceived usefulness (PU), perceived enjoyment (PE), perceived complexity (PC), perceived risk (PR), perceived value (PV), and AI of particular innovations. To ensure content validity, all items were adapted from previous research, and the wording was modified to fit internet health care services. All items of the VAM component of the model were covered by 16 questions. The questionnaire items were measured with a 5-point Likert scale ranging from “strongly disagree” (1) to “strongly agree” (5). Further details of the questionnaire are provided in Textbox 1. The items in the questionnaire were tested for multicollinearity, and the results showed that there was no multicollinearity (Table S1 in Multimedia Appendix 1).

**Measurement items of the constructs.**

**Perceived usefulness**

- I think it is convenient to use the internet health care technology (IHT) anywhere and anytime to help me serve patients.
- I think IHT can support me to serve more patients from other regions.
- I think IHT can support me in obtaining basic information about my patients in advance so that I can better respond to their health requirements.

**Perceived enjoyment**

- I believe that the use of internet health care is effective in maintaining and improving the well-being of patients.
- I believe that the use of IHT will increase my reputation and satisfaction from patients.

**Perceived** **complexity**

- I think it requires cumbersome registration, real name verifications, and login processes to obtain permission to use IHT.
- I think that there are more complex procedures and processes required to provide web-based IHT than traditional offline services.
- I think getting into the interface is a challenge when using IHT.

**Perceived risk**

- I am worried that the use of IHT without the opportunity to interview patients will increase health care risks and exacerbate doctor-patient disputes.
- I am concerned about the security of my personal information, passwords, consultation records, and other information being stolen when using IHT.
- I am worried that using IHT would consume too much time and increase my workload.

**Perceived value**

- I think there are more benefits to using IHT than the effort expended on it.
- I think there are more benefits to be gained from using IHT than risks that may be encountered.

**Adoption intention**

- I will try to use or continue to use IHT.
- I would like to recommend IHT to those who require it.
- In the future, I will use IHT frequently.

#### Employee Burnout

Burnout among health care professionals was measured by the Maslach Burnout Inventory-General Survey (MBI-GS) [30]. The MBI-GS consists of 15 items over 3 metrics: emotional exhaustion, cynicism, and reduced professional efficacy. The items are scored on a Likert scale from “never” (0) to “every day” (7). The MBI-GS does not compute a total score for burnout to reflect the burnout state. The higher the scores on the 3 metrics, the higher the level of burnout indicated. The Chinese version of the MBI-GS, developed by Li [79], also has good validity and reliability, obtaining a Cronbach α of .920. In addition, the factor loadings of the measured items were higher than 0.72, indicating a reasonable construct validity of the scale.

The preliminary design of the questionnaire was reviewed by experts familiar with the research topic to ensure that the questions successfully captured the topic and did not contain common errors, such as leading, confusing, or double-barreled questions. Prior to the survey, a pilot study was conducted among 20 health care professionals to ensure that there were no problems in reading the frame information and understanding and answering the questions. All participants said that the frames were easy to understand, and the length of the questionnaire was appropriate.

#### Participants

**Figure 1:**
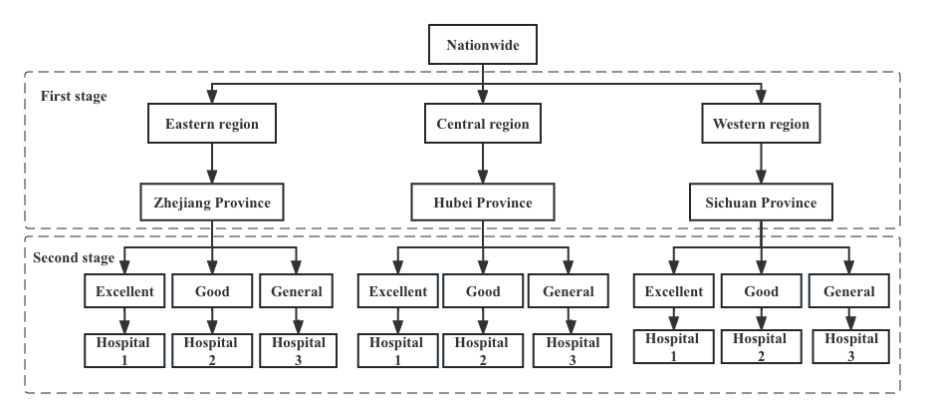
The sampling method of this study.

This study was conducted using a multistage sampling method to ensure that the sample was representative. In the first stage, a stratified random sampling method was used to divide the 23 provinces and 5 autonomous regions of China according to geographical region (eastern, central, and western regions) and economic development, with 1 provincial administrative region randomly selected. In the second stage, 1 tertiary public hospital was randomly selected as a sample hospital within each rank of the provincial administrative regions according to the performance appraisal of tertiary public hospitals in 2019 (excellent, good, and general). The 3 representative hospitals were located in each of the provinces. All hospitals’ health care professionals were included, excluding workers not involved in health care, such as administration and logistics workers. The health care professionals were involved in clinician, nursing, public health, medical technology, pharmacy, etc. The sampling method is illustrated in Figure 1.

The questionnaire was accompanied by detailed instructions and informed consent. All questions were required to be completed before submission. In addition, the same IP could only be filled once to combat duplicate completion. We also set a time restriction on completing the questionnaire. All questionnaires that were answered in less than 1 minute were also excluded. Moreover, the same questions with the same answers for consecutive questions were excluded. Finally, 12,031 valid questionnaires were obtained.

### Ethics Approval

This study received ethical approval from the Ethics Committee of the School of Public Health, Shanghai Jiao Tong University School of Medicine, China (SJUPN-202008).

### Hypotheses Development and Control Variable

**Figure 2:**
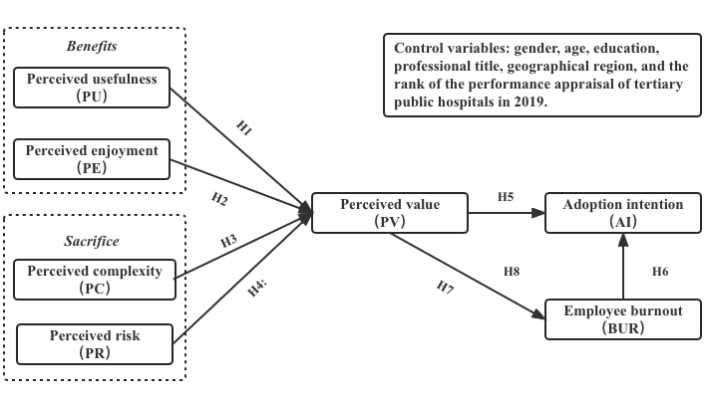
Theoretical model: research framework combining value-based adoption model and employee burnout to predict the adoption of internet health care technology from the perspective of health care professionals. H: hypothesis.

This study proposes 8 research hypotheses based on the VAM model and burnout theory (Figure 2).

- H1: Perceived usefulness is positively related to perceived value.
- H2: Perceived enjoyment is positively related to perceived value.
- H3: Perceived technicality is negatively related to perceived value.
- H4: Perceived risk is negatively related to perceived value.
- H5: Perceived value is positively related to adoption intention.
- H6: Employee burnout is negatively related to adoption intention.
- H7: Perceived value is negatively related to employee burnout.
- H8. Employee burnout mediates the relationship between perceived value and adoption intention (see Multimedia Appendix 1 for more details about literature review and hypotheses development).

Adding control variables will increase the explanatory power of this work [80]. Thus, we include the control variables of gender, age, education, professional title, geographical region, and the rank of the performance appraisal of tertiary public hospitals in 2019.

### Statistical Analysis

Descriptive analysis was performed to summarize participants’ sociodemographic characteristics after the data were exported to SPSS (version 23.0; IBM Corp). The extent of relationships among variables were evaluated by structural equation modeling using SPSS Amos version 23. Cronbach α was used to check the reliability and the verification standard was α>.7. Confirmatory factor analysis was used to discuss the structural validity and internal consistency of each construct; the average variance extracted (AVE) values were used to measure the correlation between different structures [81,82]. All hypotheses from the studies were also tested. Assumptions of multivariate normality, multicollinearity, sample size appropriateness, and positive definiteness were then checked.

## Results

### Demographic Characteristics

In this study, 9453 (78.6%) respondents were female, 2792 were clinicians, 5200 (43.2%) respondents were between the ages of 30-39 years, 8537 (71%) respondents had received a college education, and half of the respondents were from eastern China. Table 1 provides further details. We also found that 6055 (50.3%) health care professionals came from the eastern coastal provinces in this survey. This may be related to the economically developed and abundant health care resources in the eastern part of China.

**Table 1:** Demographics of respondents.

Characteristic		Respondents, n (%)
**Gender**		
	Male	2578 (21.4)
	Female	9453 (78.6)
**Age (years)**		
	<30	4330 (36.0)
	30-39	5200 (43.2)
	40-49	1717 (14.3)
	≥50	784 (6.5)
**Professional background**		
	Clinician	2792 (23.2)
	Nurse	7220 (60.0)
	Public health	47 (0.4)
	Medical technician	1472 (12.2)
	Pharmacist	500 (4.2)
**Education level**		
	Doctoral degree	559 (4.6)
	Master’s degree	1554 (12.9)
	Bachelor’s degree	8537 (71.0)
	High school graduate	1267 (10.5)
	Less than high school	114 (0.9)
**Location**		
	West region	2263 (18.8)
	Central region	3713 (30.9)
	East region	6055 (50.3)
**Professional title**		
	Senior	445 (3.7)
	Vice senior	1047 (8.7)
	Intermediate	3988 (33.1)
	Junior	5666 (47.1)
	None	885 (7.4)
**Performance**		
	Excellent	4846 (40.3)
	Good	5526 (45.9)
	General	1659 (13.8)

### Reliability and Validity Test

A confirmatory factor analysis was performed to test the measurement equation, including reliability and validity tests. As presented in Table 2, the reliability and convergent validity of items and constructs are assessed using Cronbach α, composite reliability, and AVE. Constructs with Cronbach α and composite reliability value >0.7 and AVE>0.5 are considered acceptable. Convergent validity is established by evaluating composite reliability and AVE. Accordingly, all constructs demonstrate an acceptable level of reliability and validity. Item loadings range from 0.716 to 0.916 and are all significant at the .01 level. Composite reliabilities range from 0.838 to 0.925, and AVE ranges from 0.642 to 0.806. Combined, these indices indicate a high degree of convergent validity. Cronbach α coefficients range from 0.843 to 0.926, which suggest a high level of reliability.

The discriminant validity was conducted to evaluate the range to which a provided study latent variable is distinct from others. Hence, when the average variance extracted of an individual latent construct is higher than the multiple squared correlations of that construct with other constructs, the discriminant validity will be at an acceptable level. The discriminant validity was tested using the Fornell-Larcker criterion. The square roots of the corresponding AVE are on the diagonal, where each construct’s AVE is higher than its highest correlation with any other construct. The results are shown in Table 3.

Fit measures of the structural model are presented in Table 4. The values of goodness-of-fit index, adjusted goodness-of-fit index, comparative fit index, and normed fit index are greater than 0.90. The fit indices of the study models are all higher than the normal average acceptance level, which indicates a good fit of the study models to the collected data.

**Table 2:** Reliability and validity.

Constructs and items		Standard loading	Cronbach α	Mean (SD)	AVE^a^	CR^b^
**PU^c^**			.897		0.743	0.897
	PU1	0.817		3.799 (0.765)		
	PU2	0.879		3.915 (0.703)		
	PU3	0.896		3.896 (0.698)		
**PE^d^**			.876		0.780	0.876
	PE1	0.907		3.913 (0.685)		
	PE2	0.861		3.849 (0.724)		
**PC^e^**			.838		0.643	0.843
	PC1	0.718		3.449 (0.910)		
	PC2	0.858		3.258 (0.933)		
	PC3	0.819		3.080 (0.925)		
**PR^f^**			.842		0.642	0.843
	PR1	0.835		3.526 (0.857)		
	PR2	0.805		3.521 (0.862)		
	PR3	0.767		3.258 (0.930)		
**PV^g^**			.854		0.746	0.854
	PV1	0.851		3.549 (0.768)		
	PV2	0.877		3.565 (0.759)		
**AI^h^**			.925		0.806	0.926
	AI1	0.878		3.874 (0.649)		
	AI2	0.916		3.933 (0.656)		
	AI3	0.900		3.894 (0.686)		

^a^AVE: average variance extracted.

^b^CR: composite reliability.

^c^PU: perceived usefulness.

^d^PE: perceived enjoyment.

^e^PC: perceived complexity.

^f^PR: perceived risk.

^g^PV: perceived value.

^h^AI: adoption intention.

**Table 3:** Discriminant validity.

	PU^a^	PE^b^	PC^c^	PR^d^	PV^e^	AI^f^
PU	0.862	N/A^g^	N/A	N/A	N/A	N/A
PE	0.848	0.883	N/A	N/A	N/A	N/A
PC	0.065	0.099	0.802	N/A	N/A	N/A
PR	−0.043	−0.029	0.617	0.801	N/A	N/A
PV	0.569	0.589	0.196	0.049	0.864	N/A
AI	0.692	0.704	0.074	−0.019	0.618	0.898

^a^PU: perceived usefulness.

^b^PE: perceived enjoyment.

^c^PC: perceived complexity.

^d^PR: perceived risk.

^e^PV: perceived value.

^f^AI: adoption intention.

^g^N/A: not applicable.

**Table 4:** Model fit indices.

Index	Model value	Recommended value	Acceptance
RMSEA^a^	0.065	<0.05 good fit and <0.10 reasonable fit	Reasonable
GFI^b^	0.928	Above 0.9	Good
AGFI^c^	0.902	Above 0.9	Good
TLI^d^	0.922	Above 0.9	Good
CFI^e^	0.937	Above 0.9	Good
NFI^f^	0.936	Above 0.9	Good

^a^RMSEA: root mean square error of approximation.

^b^GFI: goodness-of-fit index.

^c^AGFI: adjusted goodness-of-fit index.

^d^TLI: Tucker-Lewis index.

^e^CFI: comparative fit index.

^f^NFI: normed fit index.

### Structural Model Testing

**Figure 3:**
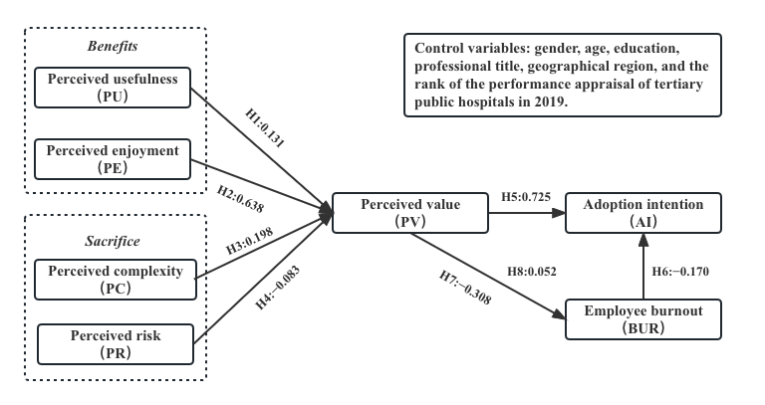
Results of the final research model. H: hypothesis.

Figure 3 shows that all 7 (H1-H8) research hypotheses are supported. Table 5 indicates the total, direct, and indirect effect of the model variables on attitude toward the AI. PE had the strongest positive relationship with PV (β=.638, P<.001). PU had a moderate positive relationship with PV (β=.131, P=.01). Furthermore, PR had a slightly negative direct relationship with PV (β=−.083, P<.001). Surprisingly, PC had a moderately positive relationship with PV (β=.198, P<.001). PV had a negative relationship with BUR (β=−.308, P<.001), BUR had a negative effect on AI (β=−.170, P<.001), and PV had a positive direct effect on AI (β=.725, P<.001). In addition, PV had an indirect effect on AI, and the effects were mediated by BUR (β=.052, P<.001).

**Table 5:** Path decomposition of effects in structural equation modeling.

	Direct effect	P value	Indirect effect	P value	Total effect	P value
PU^a^→PV^b^	0.131	.01	N/A^c^	N/A	0.131	.01
PE^d^→PV	0.638	<.001	N/A	N/A	0.638	<.001
PC^e^→PV	0.198	<.001	N/A	N/A	0.198	<.001
PR^f^→PV	−0.083	<.001	N/A	N/A	−0.083	<.001
PV→BUR^g^	−0.308	<.001	N/A	N/A	−0.308	<.001
BUR→AI^h^	−0.170	<.001	N/A	N/A	−0.170	<.001
PV→AI	0.725	<.001	0.052	<.001	0.778	<.001

^a^PU: perceived usefulness.

^b^PV: perceived value.

^c^N/A: not applicable.

^d^PE: perceived enjoyment.

^e^PC: perceived complexity.

^f^PR: perceived risk.

^g^BUR: employee burnout

^h^AI: adoption intention.

## Discussion

### Principal Findings

China is now facing an aging population, which has resulted in an expansion of the chronic disease patient population and has substantially increased the demand for health care resources. However, the “siphon effect” of high-quality public hospitals and the inappropriate allocation of health care resources have led to a growing problem of “difficult and expensive access to health care,” as well as a serious imbalance between supply and demand for health care services. In this context, the integration of information technology and health care has accelerated, and the internet health care service model has emerged to satisfy the public demand for life cycle health care services. In addition, the COVID-19 pandemic has created significant development opportunities for internet hospitals since 2020. Based on the need for pandemic prevention and control in China, the National Health Commission, the National Health Insurance Bureau, and other health departments have established a series of policies to vigorously promote the development of internet hospitals [83-85]. Engaging more health care professionals in internet health care is necessary to realize the healthy and sustainable development of internet hospitals. However, Lo et al [86] found that the overall prevalence of burnout symptoms among doctors in China ranged from 66.5% to 87.8%, with young doctors and doctors working in tertiary hospitals being more at risk of burnout. The deterioration of the doctor-patient relationship has become a major problem in China’s health care system, with one-third of doctors experiencing conflict and thousands being injured [87]. Burnout for health care professionals may influence their engagement in internet health care. This study first integrates VAM and burnout theory to explain the AI toward IHT. The 2 perspectives are then used to propose a research model for exploring the adoption of mobile health care services in a complementary manner. The primary findings are summarized as follows.

First, the results indicate that PU and PE have a strongly positive effect on PV. Moreover, PE has a much greater influence on PV than PU (β=.638 vs .131). PV has a strongly positive direct effect on AI toward IHT, and the findings are consistent with the work of Wu et al [31]. Most prior research focused on the factors of perceived ease of use, PU, and social support value, which could facilitate individuals’ IHT adoption [88]. In this study, PE was found to be particularly important in encouraging PV. The reason behind this may be due to health care professionals being more concerned with improving the doctor-patient relationship and increasing satisfaction and reputation among patients than facilitating service to patients [80]. This further reflects the tension between doctors and patients in China. Therefore, it would be useful for IHT providers to offer more practical benefits in order to attract more health care professionals and retain existing ones, including “reputation rewards,” “answer rating,” and other incentives, to help health care professionals build their reputation and customer satisfaction, which also contributes to enhancing the reputation of internet hospitals [32,80,89].

Second, according to our findings, PR can inhibit health care professionals’ AI. The PRs in this study included privacy risks concerning personal information, passwords, consultation records, and other information used in IHT. Other studies showed that privacy risk is an important factor in the decline of willingness to use IHT, and Guo et al [90] and Zhang et al [91] concluded that privacy risk could affect AI toward IHT. Although prior research has examined the factors that can inhibit individuals’ IHT adoption, most research focuses on the factors of privacy risk, performance risk, and legal concerns [33,90,91], ignoring workload and doctor-patient dispute problems. This study extends the results of prior studies by demonstrating that workload and doctor-patient disputes are significant inhibitors of IHT AIs. Hence, IHT providers should improve the overall quality and use security protection mechanisms to ensure the security of users’ private information [33]. Meanwhile, an appointment model can be adopted to reduce the workload and improve the adoption enthusiasm of health care professionals.

To our surprise, we found that PC positively affects PV (β=.198). In general, PC has a negative influence on PV. Currently, the recent health care environment is involved in a major shift in internet technology updates, with increasing technological complexity and the management of more patients with fewer resources, thus health care practitioners are faced with higher requirements [92]. Within the technological context, complexity explains how the new technology is perceived in terms of its use and comprehension by the users. In Malaysia, Ahmadi et al [93] surveyed the experts’ adoption of a hospital information system within public hospitals. They found that PC of hospital information system innovation leads to resistance due to the lack of skills and knowledge. However, we found that PC positively affects PV. In Saudi Arabia, Alamri [94] investigated students’ adoption of massive open online courses (MOOC) during the COVID-19 pandemic. They found PC was positively connected with perceived ease of use and PU. They thought that if the MOOC systems were able to satisfy students’ research needs, notwithstanding the PC, they will also consider the MOOC system to be helpful during the COVID-19 pandemic. Complicated operations in health care work are designed to provide greater protection for the privacy of doctors and patients and safeguard the security of their accounts, such as registration, name verification, and log-in processes. In addition, individuals are often more technologically literate than their peers 10 years ago and can easily adapt to modern mobile health services [34]. Moreover, 9530 (79.2%) health care professionals in this study were younger than 40 years, which is similar to the percentage reported in the China Health Statistics Yearbook 2020. These health care professionals are more likely to adopt IHT. The younger generations of health care professionals are more accepting of the newest internet technologies. This also may be due to health care professionals considering the value gained from internet health care as greater than the effort expended. Even so, IHT providers should improve service quality and simplify processes for health care professionals to enhance adoption.

Third, this study confirmed that BUR is negatively related to AI (β=−.170), the result is consistent with the findings of other studies. Gardner et al [26] reported that 70% of clinicians experienced health information technology–related stress. Physicians reporting moderately high or excessive time spent on electronic health records at home were 1.9 times more likely to experience burnout [90]. A qualitative study found that electronic consultations shifted some burden of specialty work to primary care clinicians. Electronic consultations can contribute to burnout through increased administrative burden and changing workflow for providers, especially in the immediate term [26,95]. In addition, we found that the PV inhibits BUR (β=−.308). PV also has indirect effects on AI, and the effects are mediated by BUR (β=.052). Hartzband et al [96] reported that autonomy, competence, and relatedness supported the restoration of intrinsic motivation and the reversal of burnout in health care professionals. Moreover, they deemed that doctors wanted to give patients the time and support they needed and wanted the system to value and recognize their efforts to provide this kind of health care. This perspective is consistent with the findings of our study. Furthermore, the tense doctor-patient relationship in China has resulted in low patient respect for health care professionals, resulting in burnout [97]. Nevertheless, the original motive that drives a doctor to endure hardship is to relieve the suffering of patients. Hence, the potential strategies for managing burnout in Chinese health care professionals should consider promoting the medical humanities and restoring moral respect for doctors [86]. IHT providers must also undertake measures to increase the PV of health care professionals so that they feel that the benefits of engagement in internet health care outweigh their efforts.

### Limitations

This study has several limitations. First, the data we collected were obtained from the health care professionals of tertiary public hospitals in China. From a perspective of data integrity, data from health care professionals in primary hospitals should also be included. Second, this study focused on the facilitators and the inhibitors of AI in health care professionals. There are other potential factors that may influence AI that should be explored in future research. Third, we did not analyze the internet technology, such as application scenarios and the functionality of use, only the AI to use the technology. Fourth, the ratio of doctors-nurses among the health care professionals surveyed in this research was 1:2.59, which may deviate from the actual situation. Therefore, the ratio of doctors to nurses to medical technicians should be fully considered in future studies. Fifth, it was found that burnout has a negative effect on the AI of IHT. It is hoped that further research will reveal the mechanisms to explain the negative relationship between burnout and AI of IHT.

### Conclusions

This research aimed to determine the key factors influencing the AI of IHT among health care professionals based on the VAM and BUR theory. The results indicated that PU, PC, and PE positively correlated with AI via PV. In contrast, PR negatively correlated with PV, and PV negatively correlated with BUR. BUR mediated the relationship between PV and AI. These findings provide valuable information to internet health care service system designers, governments, investors, and hospital administrators to promote the use of this technology by health care professionals.

## Appendix

Multimedia Appendix 1 Literature review and hypotheses development.

## Abbreviations

AI: adoption intention
AVE: average variance extracted
BUR: employee burnout
IHT: internet health care technology
MBI-GS: Maslach Burnout Inventory-General Survey
PC: perceived complexity
PE: perceived enjoyment
PR: perceived risk
PU: perceived usefulness
PV: perceived value
VAM: value-based adoption model
