# Supplementary material for: Determinants Influencing the Adoption of Internet Health Care Technology Among Chinese Health Care Professionals: Extension of the Value-Based Adoption Model With Burnout Theory
Source: J Med Internet Res. 2023 Mar 10;25:e37671. doi: 10.2196/37671 (PMC10039406; doi:10.2196/37671)
Supplement: Multimedia Appendix 1 [file jmir_v25i1e37671_app1.docx]

**Multimedia Appendix 1**

**Literature Review and** **Hypotheses Development**

Much has been made of the potential for IHT to offer convenience, low cost and ease of access to health-related information and communications. In doing so, Internet healthcare can help patients to become more involved in their decision making and increase their autonomy. COVID-19 has been attributed with providing a catalyst to accelerate the adoption of this technology driven by the need to offer care at a distance [14]. Some studies capture how Internet healthcare has played a crucial role during the COVID-19 pandemic by helping doctors to access patients' disease histories, and to enable health education and health management for patients [35-36]. In China, Internet hospitals provided various services during the COVID-19 pandemic, including medical prescription, drug delivery, and medical insurance services [7]. In addition, Xie et al. found that many Internet hospitals were not yet mature and faced various issues, such as online doctor scarcity and the unavailability of health insurance coverage [8]. Online diagnosis and treatment continue to experience challenges in terms of resource allocation and industry supervision [37].

A variety of factors are associated with the adoption of such technology. Almojaibel et al. for example found that perceived usefulness as an important factor associated with health care providers' intent to use telerehabilitation for pulmonary rehabilitation [38]. Klingb et al. used the technology acceptance model (TAM) to identify factors that influence health care provider's attitudes toward mHealth technology for emergency care of burn patients. They found a significant relationship between compatibility and usefulness in shaping attitudes [39]. Saigi-Rubió F et al. also used the technology acceptance model (TAM) to provide evidence on the determinants of telemedicine use in clinical practice finding that security, confidentiality, reduced cost, the patients' predisposition were the most important influencing factors [40]. Other research includes that of Zhou who found that medical service satisfaction, ease of use, and information quality had a significant impact on elderly patients’ acceptance of telehealth, and acceptance had a significant impact on the elderly patients’ behavior intentions according to the technology acceptance model [41]. Alharbi highlighted the importance of facilitating conditions and trust factors for healthcare consumers on digital healthcare platforms, especially during the COVID-19 pandemic [42].

While these and many other contributions highlight the various factors shaping the adoption of Internet healthcare, less has been made of the experience of healthcare professionals in their interactions with IHT. Of course, healthcare professionals had to consider healthcare burnout while embracing new technology, and there have been several studies that have shown that the use of new technology can exacerbate employee burnout. Moreover, burnout may also reduce the adoption of intention to use IHT [21-29]. And will the update of IHT relieving or exacerbating the burden on healthcare professionals? Will burnout have an impact on the use of IHT? Therefore, it is necessary to understand how employee burnout affects healthcare professionals’ adoption intention of IHT, both positively and negatively.

The technology adoption intention of users has attracted significant attention in the field of Internet healthcare. Many models and theories can be used to study user behavior, such as the technology acceptance model, rational behavior theory, planned behavior theory, value-based adoption theory, and the UTAUT. Value-based adoption employs Kim's theory of consumer choice and decision-making from economics and marketing research [43]. The VAM has been used to analyze new services resulting from the development of information technology [34, 44-45]. This model is based on perceived benefits and perceived sacrifices to explain the adoption intention of individuals using technology. Benefits are separated into enjoyment and usefulness based on cognitive evaluation theory (CET), while sacrifices include the sub-dimensions of technicality (cost in time and effort) and fees (monetary cost). Usefulness and enjoyment have been shown to influence adoption decisions by healthcare professionals in healthcare-specific contexts [31, 46-47]. Technicality, workload, and cost are considered, particularly reduced information security. The VAM has been combined with research models to identify intentions toward Internet use [48] and the deployment of online-to-offline services.

### Hypotheses and Research Model

As shown above, research on healthcare professionals’ adoption intention of IHT has several gaps, specifically when describing what variables influence these behaviors. Therefore, we develop a new model using VAM and employee burnout as the framework, and identify relevant variables based on the literature.

#### Perceived usefulness (PU)

According to Holden and Karsh, perceived usefulness refers to one’s subjective perception that the use of new technologies or services will improve one’s work efficiency [49]. Individuals evaluate the consequences of their behavior in terms of perceived usefulness and base their choice of behavior on the desirability of the usefulness. Internet medical services can benefit medical professionals by enhancing or improving their work performance. Such services might be useful for medical professionals to obtain patients’ health information easily and rapidly, thereby eventually improving the efficiency of diagnosis and treatment and increasing patient satisfaction [50-51]. Furthermore, the IHT could serve patients who are located at a long distance and could also be easily used for their follow-up visits [8, 52]. Deng et al. confirmed that perceived usefulness was positively associated with trust toward the use of mHealth services [33]. Wu et al. proved that medical staff were likely to adopt mHealth services if they perceived them useful in their daily work [31]. Kissi et al. evaluated physicians’ satisfaction with internet healthcare services adoption and found that perceived usefulness positively influenced physicians’ behavioral intentions [53]. According to the VAM theory, if one perceives a service as useful, they will evaluate it as highly positive.

H1. Perceived usefulness is positively related to perceived value.

#### Perceived enjoyment (PE)

Hsu et al. [54] defined enjoyment as the degree to which an Internet user participates in internet work because the process “yields fun and enjoyment” and suggested that enjoyment is a factor that determines the intention of users to participate in internet work. The Motivational Model [55] states that the behaviors related to computer usage are determined by both extrinsic motivation, which refers to the performance of an activity apart from its own sake, and intrinsic motivation, which refers to the performance of an activity for its own sake. According to the model, perceived usefulness is an example of extrinsic motivation, whereas perceived enjoyment is an example of intrinsic motivation. Enjoyment in helping others is defined as the perception of pleasure obtained from helping others via knowledge contribution [56]. For doctors, it is their responsibility and intrinsic motivation to maintain the health or rehabilitation of their patients [51]. Yang et al. showed that patients prefer to share treatment experiences for doctors who work in hospitals with a higher online reputation [32]~~.~~ Sweeney et al. found that the benefit component comprised perceived enjoyment, as well as perceived usefulness [57]. Teo et al. suggested that perceived enjoyment had a significant effect on Internet usage [58]. Moghavvemi S et al. showed that perceived enjoyment is a main factor effecting students sharing knowledge through Facebook [59]. Alalwan et al. found that perceived enjoyment was statistically supported to have a significant impact on the Saudi customer intention to adopt mobile internet [60]. Furthermore, enjoyment was found to have a significant effect on technology acceptance beyond usefulness.

H2. Perceived enjoyment is positively related to perceived value.

***P******e******r******ceived*** ***c******o******m******p******l******exity (PC)***

Technicality reflects the non-monetary cost component of sacrifice. Ease of use has been widely used as an element of technicality [8, 43]. And complexity is about the cost of using the technology [51]. The technology may create additional complexity because it required advanced knowledge to use it and more [61]. In the context of Internet medical services, perceived complexity refers to the degree of difficulty experienced during the use of Internet medical services, such as registration and system operation, even more complex operations may be required for system updates, increasing the cost of your own efforts [51]. Pokhrel et al. found that it was time-consuming to enter all symptoms into an app by typing when using mobile app-based medical services [62]. Ayyagari et al. showed that perceived complexity was associated with increased effort demanded by the technology. As technology becomes more complex, users may become more frustrated with the number of features and how to use them [63]. Sullivan et al. suggested that technology users are likely to discontinue using an operating system if they perceive the system is difficult to use [64].

H3. Perceived technicality is negatively related to perceived value.

***Perceived risk (PR)***

Perceived risk refers to one’s perception of uncertainty in the use of Internet medical services and its severity in terms of consequences [65]. Previous Internet related research identifies PR as an important attitudinal factor that influences adoption behavior, but PR investigation is mainly focused on transaction security risk or privacy risk [66-67]. Much research has applied PR to consumer behavior studies and further identified the multi-dimensional nature of the perceived risk construct, such as performance risk [68] “The possibility of the product malfunctioning and not performing as it was designed and advertised and therefore failing to deliver the desired benefits”, this may even increase the doctor-patient conflict in Internet healthcare [51, 69]. privacy risk [68] “Potential loss of control over personal information, such as when information about you is used without your knowledge or permission”. Health-related data is some of the most sensitive information about a person [69]. Compared with traditional health services based in hospitals, individuals are more likely to experience a loss of information privacy when using online medical services [70] and time risk [68] “Consumers may lose time when making a bad purchasing decision by wasting time researching and making the purchase, learning how to use a product or service only to have to replace it if it does not perform to expectations.” Pokhrel et al. showed that using an app increased the health practitioner’s workload instead of reducing it as they had to enter the patient’s information twice: once in the health facility’s register and again in the app [62]. Healthcare professionals may also believe that patients will have a negative perception that they are not concentrating or listening to patients due to this increase in workload.

H4. Perceived risk is negatively related to perceived value.

***Perceived value (PV)***

Perceived value is a significant factor affecting consumers’ choice to facilitate (or block) achieving their goals with their perceived preference for and evaluation of a certain product [72]. Perceived value can be defined by comparing benefits with sacrifices and is an indicator of adoption intention [43]. Chen believed that doctors would only choose to use Internet healthcare to provide care to their patients if they perceived that the benefits (in terms of money, satisfaction, etc.) provided by Internet healthcare outweighed the sacrifices (in terms of energy and time costs, etc.) required [51]. Kim et al found that perceived value was affected by both perceived benefit and perceived sacrifice in research on IoT-based smart home service [45]. In research on digital music services, Turel et al identified that higher perceived value had a positive impact on intention to use [73]. Aaker et al claimed that the higher the perceived value, the more consumers were inclined to exhibit a favorable attitude or intention to use towards a brand [74].

### *A**d**o**p**t**i**on intention (AI)*

Adoption intention has been identified as a basic requirement of the individuals' actual behaviour in adopting a new system and technology [60,75]. Particularly for sectors such as IT where new technologies are utilized, it has been verified that consumer attitude has a direct impact on intention to use [76]. Doctors will decide to provide Internet medical services for patients only when they believe that the benefits (income and popularity) are greater than the sacrifices (time, energy, etc.) [51].

H5. Perceived value is positively related to adoption intention.

***E******m******p******l******o******yee burnout (BUR)***

In 1981, Maslach and Jackson defined burnout as “a syndrome of emotional exhaustion and cynicism that occurs frequently among individuals who do ‘people work’ of some kind” [30]. As burnout evolves, the physician’s work performance deteriorates, errors are more likely, and patients may be harmed [77]. Physician burnout may also indirectly increase healthcare expenditures via higher rates of medical errors and malpractice claims due to absenteeism and lower job productivity, as previously noted [78]. Several researchers found that there was a negative relationship between technology acceptance and employee burnout [21-29].

H6. Employee burnout is negatively related to adoption intention.

H7. Perceived value is negatively related to employee burnout.

H8. Employee burnout mediates the relationship between perceived value and adoption intention.

As a result, Table 1 presents the generated hypotheses in the current study. It can be seen that the hypotheses are categorized in the six main dimensions.

Table 1. Summary of proposed hypotheses.

| **Dimension** | **Definition** | **Reference** | **Hypotheses statements** |
| --- | --- | --- | --- |
| Perceived usefulness (PU) | Perceived usefulness refers to one’s subjective perception that the use of new technologies or services will improve one’s work efficiency. | [47-53] | Perceived usefulness is positively related to perceived value. |
| Perceived enjoyment (PE) | Perceived enjoyment  is defined as the extent to which the activity of using a specific technology is perceived to be enjoyable, aside from any performance consequences resulting from technology use | [54-60] | Perceived enjoyment is positively related to perceived value. |
| Perceived complexity (PC) | Perceived complexity refers to the degree of difficulty experienced during the use of Internet medical services, such as registration and system operation | [8,43,51, 61-64] | Perceived technicality is negatively related to perceived value |
| Perceived risk (PR) | Perceived risk refers to one’s perception of uncertainty in the use of Internet medical services and its severity in terms of consequences | [51,62, 65-70] | Perceived risk is negatively related to perceived value. |
| Adoption intention (AI) | Perceived value can be defined by comparing benefits with sacrifices and is an indicator of adoption intention | [51, 60, 75-76] | Perceived value is positively related to adoption intention. |
| Employee burnout (BUR) | Employee burnout was defined as a syndrome of emotional exhaustion and cynicism that occurs frequently among individuals who do ‘people work’ of some kind | [21-29, 77-78] | Employee burnout is negatively related to adoption intention. |
| Perceived value (PV) | Perceived value is a significant factor affecting consumers’ choice to facilitate (or block) achieving their goals with their perceived preference for and evaluation of a certain product | [43, 45, 51, 72-74] | Perceived value is negatively related to employee burnout |
|  |  |  | Employee burnout mediates the relationship between perceived value and adoption intention |

**Table 1S Multi-collinearity test**

|  | Tolerance | VIF |
| --- | --- | --- |
| PU1 | .374 | 2.675 |
| PU2 | .289 | 3.457 |
| PU3 | .264 | 3.789 |
| PE1 | .258 | 3.877 |
| PE2 | .323 | 3.096 |
| PV1 | .417 | 2.398 |
| PV2 | .409 | 2.445 |
| PC1 | 0.528 | 1.893 |
| PC2 | 0.397 | 2.520 |
| PC3 | 0.418 | 2.392 |
| PR1 | 0.434 | 2.303 |
| PR2 | 0.458 | 2.186 |
| PR3 | 0.480 | 2.085 |

When the tolerance is less than 0.1 or the VIF is greater than 5 or 10, this indicates the presence of multicollinearity between the independent variables.

**Table 2S Confirmatory Factor Analysis**

| Index | Burnout Model value | VAM Model value | Recommended value | Acceptance |
| --- | --- | --- | --- | --- |
| RMSEA ^a^ | 0.055 | 0.058 | ˂ 0.05 good fit ˂ 0.10 reasonable fit | Reasonable |
| GFI ^b^ | 0.972 | 0.958 | Above 0.9 | Good |
| AGFI ^c^ | 0.954 | 0.942 | Above 0.9 | Good |
| TLI ^d^ | 0.978 | 0.966 | Above 0.9 | Good |
| CFI ^e^ | 0.984 | 0.973 | Above 0.9 | Good |
| NFI ^f^ | 0.984 | 0.972 | Above 0.9 | Good |

The factor-loadings of VAM among the measured items were from 0.71 to 0.92, indicating a reasonable construct validity of the scale.
